# High-throughput RNA dataset of viral transcripts associated with chickens from various poultry farms in North and South Kuwait

**DOI:** 10.1016/j.dib.2024.110180

**Published:** 2024-02-11

**Authors:** Salwa A. Al-Mouqatea, Vinod Kumar, Abdulmohsen Ali, Ahmed Benhejji, Sujan Eilpay

**Affiliations:** Biotechnology Program, Environment and Life Sciences Research Center, Kuwait Institute for Scientific Research, Kuwait

**Keywords:** Viral Transcriptome, High-throughput sequencing, RNA-sequencing, *Gallus gallus*, Chicken virus identification

## Abstract

Screening of poultry using RNA sequencing could enable the timely detection and identification of emerging viral pathogens, facilitating proactive measures to prevent and control potential outbreaks in the poultry industry. The reported dataset is of reads from RNA-Seq libraries consisting of approximately 130 gigabytes of RNA-sequencing data corresponding to blood, trachea and cloaca of chicken from various farms of North and South Kuwait regions. The sequences were quality-filtered and first mapped with the Chicken reference genome. The unmapped reads were aligned to the viral reference genomes. The aligned data was used for the quantification of viral transcripts across the samples. The RNA sequencing data could be useful for further meta-analysis and to extract valuable insights into the molecular landscape of poultry health. The insights gained from this research can guide the development of targeted diagnostic tools and strategies for effective poultry health management.

Specifications Table

**Table:** 

Subject	Microbiology: General; Molecular biology, Virology
Specific subject area	RNA sequencing of blood, trachea, and cloaca of chickens from various poultry farms across North and South Kuwait for the detection of viruses.
Data format	Raw data in fastq
Type of data	Table, Chart, RNA sequence
Data collection	Samples of blood, cloaca and trachea was obtained from chickens from the local farms of Kuwait. The samples were meticulously gathered from six distinct farms situated in both the northern and southern regions of Kuwait ensuring a geographically diverse dataset. Total RNA was isolated from blood, trachea, and cloacal swabs using QIAamp RNA extraction kit. Messenger RNA was purified from total RNA using poly-T oligo-attached magnetic beads. RNA was fragmented and the first strand cDNA was synthesized using random hexamer primers followed by the second strand cDNA synthesis. Samples were sequenced on an Illumina platform. Sequence data was processed using various bioinformatics tools. The high-quality filtered reads were mapped against the Chicken reference genome. The unmapped reads were aligned to the viral reference genomes. The aligned reads were quantified. The read counts were considered for comparison across samples.
Data source location	Environment and Life Sciences Research Center, Kuwait Institute for Scientific Research, Kuwait (GPS: 29.3358432, 47.9065882). Abbreviated names of the farm and their GPS coordinates as follows: AT: 28.5785041, 48.0046716; JB: 28.6361960, 48.0976860; NA: 28.5440561, 48.0846051; BY: 29.959797, 47.700319; HA: 29.269193, 47.793350; NP: 29.950589, 47.695444
Data accessibility	Repository name: NCBI Accession of SRA data: PRJNA1044791 Data identification number: PRJNA1044791 Direct URL to data: https://www.ncbi.nlm.nih.gov/bioproject/PRJNA1044791
Related research article	None

## 1 Value of the Data

• The RNA sequencing data is valuable for researchers involved in poultry transcriptome research. The dataset could be used to detect and identify emerging viral pathogens.
• The dataset offers comprehensive insights into the molecular landscape of poultry health and understanding of viral diversity for effective surveillance and risk assessment.
• The downstream analysis and interpretation of the results of the RNA sequencing data could serve as a valuable resource for researchers and provide molecular insights that support evidence-based decision-making for policymakers in the control and prevention of re-emergent viral pathogens.

## 2 Background

The escalating threat of recurrent outbreaks and the potential emergence of highly pathogenic strains of viruses necessitate a proactive and vigilant approach to monitoring viral diseases in live birds. Early detection of viral pathogens is paramount in preventing major epidemics and safeguarding both the poultry industry and public health. In this context, employing advanced molecular diagnostic tools, particularly Next Generation Sequencing (NGS) based on RNA sequencing has emerged as a crucial component in comprehensive surveillance strategies. The main objective of the study was to explore the viral transcriptome associated with Chicken tissues from six different farms in North and South Kuwait regions.

## 3 Data Description

This article describes the dataset of the linked repository of around 130 Giga bytes of RNA-sequencing data from various tissues (blood, trachea, and cloacal) of chickens from six different farms in North and South Kuwait. The sample information and the quality of the data are indicated in Tables 1 and 2, respectively.

**Table 1:** Sample information of the RNASeq libraries.

Sample	Source	Region	Farm
ATSR1B	Blood	South	AT
JBSR2B	Blood	South	JB
NASR3B	Blood	South	NA
HANR1B	Blood	North	HA
NPNR2B	Blood	North	NP
BYNR3B	Blood	North	BY
ATSR1C	Cloacal swab	South	AT
JBSR2C	Cloacal swab	South	JB
NASR3C	Cloacal swab	South	NA
HANR1C	Cloacal swab	North	HA
NPNR2C	Cloacal swab	North	NP
BYNR3C	Cloacal swab	North	BY
ATSR1T	Tracheal swab	South	AT
JBSR2T	Tracheal swab	South	JB
NASR3T	Tracheal swab	South	NA
HANR1T	Tracheal swab	North	HA
NPNR2T	Tracheal swab	North	NP
BYNR3T	Tracheal swab	North	BY

**Table 2:** Data quality summary.

Sample ID	Raw reads	Raw data	Effective (%)	Error (%)	Q20 (%)	Q30 (%)	GC (%)
HANR1B	72468502	1.09E+10	90.86	0.03	93.98	88.28	50.24
NPNR2B	81493648	1.22E+10	97.24	0.03	96.48	91.86	52.91
BYNR3B	80875058	1.21E+10	96.5	0.03	96.09	91.45	55.23
ATSR1B	72827166	1.09E+10	97.03	0.03	96.27	91.51	51.26
JBSR2B	81727634	1.23E+10	94.2	0.03	94.1	88.55	46.7
NASR3B	2.06E+08	3.09E+10	98.23	0.03	97.57	93.81	51.95
HANR1T	2.19E+08	3.29E+10	93.71	0.03	96.65	92.55	50.88
ATSR1T	93397242	1.4E+10	97.84	0.03	96.33	91.6	50.92
NASR3T	97776748	1.47E+10	89.18	0.03	94.3	89	53.1
HANR1C	77081662	1.16E+10	94.73	0.03	95.37	90.47	50.16
NPNR2C	81015468	1.22E+10	94.28	0.03	94.93	89.71	48.3
ATSR1C	70257178	1.05E+10	93.32	0.03	95.19	90.18	52.25
JBSR2C	81454326	1.22E+10	98.36	0.03	97.05	92.8	55.96
NASR3C	82781850	1.24E+10	97.45	0.03	97.26	93.13	55.12
JBSR2T	1.05E+08	1.57E+10	85.76	0.03	93.67	88.5	57.9
NPNR2T	74654236	1.12E+10	87.98	0.03	93.72	88.16	50.08
BYNR3T	70486070	1.06E+10	96.49	0.03	96.54	92.15	58.84
BYNR3C	70026768	1.05E+10	95.04	0.03	97.3	93.03	59.31

Raw reads: the total amount of reads of raw data, and equals the amount of read1 and read2; Raw data: (Raw reads) * (sequence length), calculating in G; Effective: (Clean reads/Raw reads)*100%; Error: base error rate; Q20, Q30: (Base count of Phred value > 20 or 30) / (Total base count); GC: (G & C base count) / (Total base count).

**Fig. 1:**
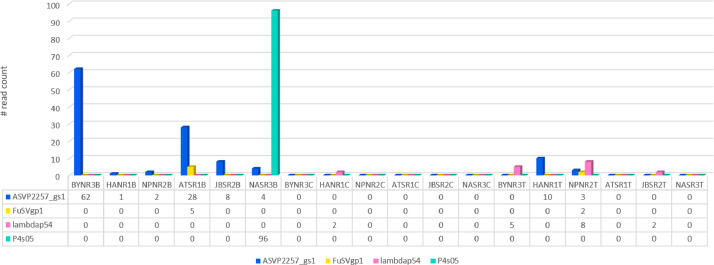
Predominant group of viruses found in the poultry RNA samples.

High-quality reads obtained after a thorough filtering process were processed further as described in the experimental design, materials and methods section to identify the viral transcripts in the samples. The read counts of identified viruses per sample are summarized in a bar-plot presented in Fig. 1.

Four viral transcripts viz., ASVP2257_gs1, FuSVgp1, lambdap54, and P4s05 corresponding to four viral species were identified that were distributed across the samples. ASVP2257_gs1 transcript from Y73 Avian sarcoma virus (PR2257 / PR2257/16) was identified in more samples (8/18) compared to others. Interestingly, this transcript is detected in all the blood samples from both North and South Kuwait farms, and tracheal swabs from two farms of North Kuwait. Another avian sarcoma viral transcript viz., FuSVgp1 from Fujinami sarcoma virus was detected in one blood (AT, South Kuwait) and one tracheal swab (NP, North Kuwait) samples. The next common transcript detected in four samples belonged to Escherichia phage Lambda (nat-host: Escherichia coli) and was detected in three tracheal swab samples of both Northern and Southern farms, and a cloacal swab sample (HA, North Kuwait). P4s05 from Enterobacteria-phage-P4 was found to be in high abundance comparatively, however it was detected only in one of the blood samples (NA, South Kuwait). Overall, across all samples, the abundance of the detected viral transcripts was low in most cases.

## 4 Experimental Design, Materials and Methods

### 4.1 Sampling

Samples of blood, cloaca, and trachea were obtained from chickens from the local farms of Kuwait. Out of the six selected farms, three were chosen from the northern region and the remaining three were selected from the southern region of Kuwait ensuring a geographically diverse dataset. The samples were coded to protect the identity of the farms. Total RNA was isolated from chicken samples using QIAamp RNA extraction kit. RNA isolated from three randomly selected samples are combined to create a composite sample for each sample type. After the pooling of triplicate samples, a total of 18 composite samples representing six farms, and three sources (blood, cloaca & trachea) from two zones (north and south of Kuwait) were selected for downstream processing. Messenger RNA was purified from total RNA using poly-T oligo-attached magnetic beads. RNA was fragmented and the first strand cDNA was synthesized using random hexamer primers followed by the second strand cDNA synthesis. Samples were sequenced on an Illumina platform. Sequence data was processed using various bioinformatics tools. The high-quality filtered reads were mapped against the Chicken reference genome. The unmapped reads were aligned to the viral reference genomes. The aligned reads were quantified. The read counts were considered for comparison across samples. Details of the analysis are provided in the subsequent sections.

### 4.2 RNA sequencing

Messenger RNA was purified from total RNA using poly-T oligo-attached magnetic beads. After fragmentation, the first strand cDNA was synthesized using random hexamer primers followed by the second strand cDNA synthesis. After end repair, A-tailing, adapter ligation, size selection, amplification, and purification, the library was checked with Qubit and real-time PCR for quantification and bioanalyzer for size distribution detection. Quantified libraries were pooled and sequenced on Illumina platforms, according to effective library concentration and data amount. Raw paired-end reads of length 150 bp were generated.

### 4.3 Bioinformatics analysis

The raw data was checked for quality before and after trimming, using FastQC v0.10.1 [1]. The raw data was trimmed for low-quality reads based on base-quality scores and read length, using TrimGalore Version 0.6.5 [2]. The minimum quality score of 20 and minimum read length of 50 were considered for trimming the raw reads. The quality filtered reads were mapped against the Chicken reference genome (GRCg7b, https://www.ncbi.nlm.nih.gov/datasets/genome/GCF_016699485.2/) using a splice aware aligner, STAR 2.7.11a [3] with default parameters. Only reads that passed the quality filters (average Phred quality score threshold of 20) were processed during the alignment. The unmapped reads were aligned to NCBI RefSeq viral reference genomes (https://www.ncbi.nlm.nih.gov/datasets/genome/?taxon=10239&annotated_only=true&refseq_annotation=true) that included around 14,000 sequences, using bowtie2 [4]. The aligned reads were quantified using HTseq 2.0.4 [5]. The read counts were considered as a quantification matrix for comparison across samples. The read counts per samples are summarized in a bar-plot presented in Fig. 1.

## Limitations

Not applicable*.*

## Ethics Statement

The authors have read and follow the ethical requirements for publication in Data in Brief and confirm that the current work does not involve human subjects, animal experiments, or any data collected from social media platforms.

## CRediT authorship contribution statement

**Salwa A. Al-Mouqatea:** Project administration, Conceptualization, Methodology, Writing – review & editing, Supervision, Funding acquisition. **Vinod Kumar:** Supervision, Validation, Methodology, Writing – original draft, Resources. **Abdulmohsen Ali:** . **Ahmed Benhejji:** . **Sujan Eilpay:** Methodology.

## Data Availability

RNA sequencing of poultry samples from six different farms of north and south Kuwait (Original data) (NCBI).
